# Analysis of miRNAs and Their Targets during Adventitious Shoot Organogenesis of *Acacia crassicarpa*

**DOI:** 10.1371/journal.pone.0093438

**Published:** 2014-04-09

**Authors:** Weina Liu, Wangning Yu, Lingyu Hou, Xiaoyu Wang, Fei Zheng, Weixuan Wang, Di Liang, Hailun Yang, Yi Jin, Xiangming Xie

**Affiliations:** College of Biological Sciences and Technology, Beijing Forestry University, Beijing, PR China; East Carolina University, United States of America

## Abstract

Organogenesis is an important process for plant regeneration by tissue or cell mass differentiation to regenerate a complete plant. MicroRNAs (miRNAs) play an essential role in regulating plant development by mediating target genes at transcriptional and post-transcriptional levels, but the diversity of miRNAs and their potential roles in organogenesis of *Acacia crassicarpa* have rarely been investigated. In this study, approximately 10 million sequence reads were obtained from a small RNA library, from which 189 conserved miRNAs from 57 miRNA families, and 7 novel miRNAs from 5 families, were identified from *A. crassicarpa* organogenetic tissues. Target prediction for these miRNAs yielded 237 potentially unique genes, of which 207 received target Gene Ontology annotations. On the basis of a bioinformatic analysis, one novel and 13 conserved miRNAs were selected to investigate their possible roles in *A. crassicarpa* organogenesis by qRT-PCR. The stage-specific expression patterns of the miRNAs provided information on their possible regulatory functions, including shoot bud formation, modulated function after transfer of the culture to light, and regulatory roles during induction of organogenesis. This study is the first to investigate miRNAs associated with *A. crassicarpa* organogenesis. The results provide a foundation for further characterization of miRNA expression profiles and roles in the regulation of diverse physiological pathways during adventitious shoot organogenesis of *A. crassicarpa*.

## Introduction

With the characteristics of rapid growth, high pulp yield, high fiber quality and ability to thrive in degraded soils [1], *Acacia crassicarpa* has become an important economic species of the Fabaceae family, and has been planted for reforestation, reclamation of wasteland, and industrial material production on a large scale in Southeast Asia [2]–[3]. However, its recalcitrance of regeneration, long life cycle, and the prolonged period needed for evaluation of traits at maturity strongly limit traditional breeding programs for the species. Development of an effective propagation strategy is required urgently.

Plant organogenesis in *vitro* is an efficient technique for large-scale propagation and popularization of selected genotypes. The technology has been extensively applied in plant genetic engineering, double haploid or polyploid breeding, and asexual reproduction of mutants or threatened species [4]–[5], for example. However, it shows strong species dependence and genotype specificity. For example, *Arabidopsis thaliana* and *Nicotiana tabacum* are easily induced to generate adventitious shoots or roots [6]–[7], whereas some plant species are recalcitrant to plant regeneration *in vitro*, such as *Triticum aestivum*, *Zea mays*, and *Gossypium* spp [8]. The regeneration frequency of leguminous trees may be quite poor in natural habitats [9]. Therefore, a thorough understanding of the molecular mechanisms and gene regulatory networks involved in organogenesis of leguminous trees is essential in order to achieve improved *in vitro* plant regeneration and genetic transformation frequencies. Although important results have been obtained regarding the hormonal regulation of organogenesis and organ-related expression of genes and proteins in leguminous trees, few studies have focused on identification and expression of microRNAs (miRNAs) during organogenesis.

MiRNAs are small endogenous non-coding RNA usually associated with gene silencing by guiding cleavage of complementary mRNAs or suppression of translation [10]. The importance of miRNAs has been realized owing to their wide occurrence in all kinds of organisms and their important biological function in regulation of gene expression [11]–[12]. Increasing evidence shows that the length of 20–24 nt miRNAs plays a crucial role in developmental and physiological processes, including developmental timing, tissue-specific development, and stem cell maintenance and differentiation [13]–[14]. Plant organogenesis and somatic embryogenesis are the most important strategies for *in vitro* plant regeneration [15]. During the last decade, several reports have demonstrated the crucial roles played by miRNAs during somatic embryogenesis. Thus, miRNA-mediated modulation of somatic embryogenesisis is closely associated with the plant regeneration process [16]–[17]. Plant organogenesis, which is also strongly associated with cell differentiation, undoubtedly is affected by miRNA repression. However, few correlative studies of miRNA function in the plant organogenesis process have been reported, especially with regard to miRNA of *A. crassicarpa*.

In order to study the regulatory role of miRNAs in organogenesis of *A. crassicarpa*, high-throughput sequencing technology ntegrated with bioinformatic analysis was employed to identify conserved and novel miRNAs involved in adventitious shoot organogenesis of *A. crassicarpa*, followed by prediction of *A. crassicarpa* miRNA targets and functions. The expression patterns of one novel and 13 conserved miRNAs during the six developmental stages of *A. crassicarpa* organogenesis were comparatively analyzed. This study is the first report focused on the modulatory roles of miRNAs during adventitious shoot organogenesis in *A. crassicarpa*.

## Materials and Methods

### Plant Materials

**Figure 1:**
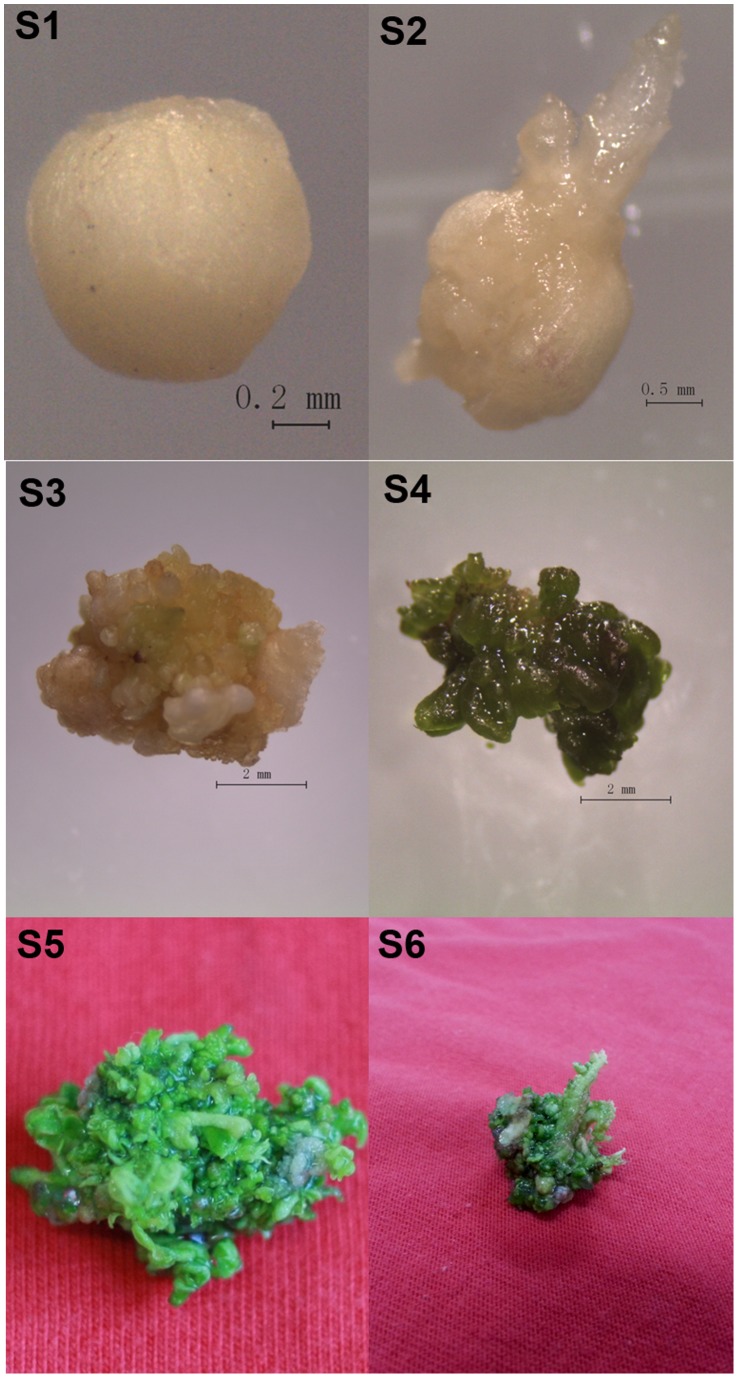
Morphology of different stages during plant organogenesis in *Acacia crassicarpa*. S1: Zygotic embryo, excised from the mature seeds. S2: Zygotic embryo differentiated, after two weeks subculture. S3: Embryogenic callus, after three weeks subculture. S4: Shoot buds, after four weeks subculture. S5: Clusters of adventitious shoots, after 5 weeks subculture. S6: Adventitious shoot elongation. S1–S4 were cultured on MS medium containing 4.54 μM TDZ and 2.85 μM IAA, observed using an Leica stereomicroscope. S5–S6 were cultured on MS medium supplemented with 2.89 μM GA_3_.

The experimental materials were obtained at different developmental stages of an adventitious bud regeneration system induced from mature zygotic embryos of *A. crassicarpa*, as described by Yao *et al.* [18](Figure 1). Based on morphological and anatomical traits, six developmental stages during shoot regeneration were defined and sampled. Generation of embryogenic callus (Figure 1, S1–S3) was induced in the dark and differentiation of shoot buds (Figure 1, S4–S6) occurred under light. All samples were frozen immediately in liquid nitrogen and stored at −80°C.

### Extraction of Total RNA, Construction of sRNA Library and Solexa Sequencing

Total RNA at the six developmental stages was extracted using the mirVana™ miRNA Isolation Kit (Ambion, Austin, TX, USA) in accordance with the manufacturer’s instructions. The quality of the isolated total RNA was assessed by analysis with a NanoDrop ND-2000 spectrophotometer (NanoDrop, Wilmington, DE, USA). The integrity of the total RNA was monitored with a Bioanalyzer 2100 and RNA 6000 Nano LabChip Kit (Agilent, Palo Alto, CA, USA). The RNA integrity number was higher than 6.0, therefore the isolated total RNA was suitable for library preparation.

Equal amounts of total RNA at each developmental stage were pooled. About 30 μg of total RNA was used for library construction and sequencing. A small RNA library was generated using the Illumina TruSeq Small RNA Sample Prep Kit (Illumina, Hayward, CA, USA) in accordance with the manufacturer’s instructions. The purified small RNA fractions between 10 and 40 nt were ligated with proprietary 5′ and 3′ adaptors separately, and then converted to cDNA by RT-PCR. The purified cDNA library was used for cluster generation on an Illumina Cluster Station and then sequenced with an Illumina GAIIx platform following the manufacturer’s instructions.

### Bioinformatic Analysis of Sequencing Data

Raw sequencing reads were obtained using Sequencing Control Studio version 2.8 software (Illumina) following real-time sequencing image analysis. The extracted sequencing reads were first processed with a proprietary pipeline script, ACGT101-miR v4.2 (LC Sciences, Houston, TX, USA), for removal of ‘impurity’ and unmappable sequences resulting from sample preparation, sequencing chemistry and processes, and the optical digital resolution of the sequencer detector. The processed raw data have been uploaded to Short Read Archive of NCBI with the accession number of SRX469994. The remaining sequences with lengths of 15 to 30 nt were mapped to precursor miRNAs (pre-miRNAs) sequences of all plants in miRBase 19.0, for all mature miRNAs included in the database. The mapping sequences were further aligned against *Acacia mangium* expressed sequence tags (ESTs) and the genomic sequence of selected plant species, including *Arabidopsis thaliana*, *Populus trichocarpa* and soybean, to identify conserved miRNAs in *A. crassicarpa*. The unmapped sequences matching sequences in other defined databases, mainly comprising mRNA, RFam, and Repbase, were removed. A BLAST search with the remaining sequences against the selected genome such as *Arabidopsis thaliana*, *Populus trichocarpa* and soybean and ESTs of *Acacia mangium* was performed, and those extended sequences at the mapped genome positions with the propensity to form hairpins (as predicted with Mfold software) were selected as putative novel miRNAs.

### Prediction of Potential Target mRNAs

Target prediction for the miRNAs was based on the principle of nearly perfect complementation between the miRNA and target mRNAs [19]. The identified conserved and putative novel miRNAs were all submitted for target gene prediction using TargetFinder (http://hercules.tigem.it/TargetFinder.html) with the default parameters, using the algorithm described by Quandt *et al* [20]. The target genes were then validated using public resources of The Arabidopsis Information Resource (TAIR; http://arabidopsis.org/). Sequences with a score of less than 4 were regarded as miRNA target genes. On the basis of their functions putative targets were classified using Gene Ontology (GO) annotations. All GO annotations were obtained from the TAIR database, and most (about 87%) of the targets were annotated. Given that many targets have more than one GO hit, the number of total hits can be higher than the total number of targets.

### Quantitative Real-time PCR

Small RNAs of *A. crassicarpa* at the six organogenesis stages were isolated using micro RNA Extraction Kit (BioTeKe, Beijing, China) following the manufacturer’s instructions. With minor modifications, poly (A)-tailed quantitative real time PCR (qRT-PCR) was chosen for experimental identification. miRNA qRT-PCR was carried out using the NCode™ VILO™ miRNA cDNA Synthesis Kit (Invitrogen, Carlsbad, USA) and SYBR Premix Ex Taq™ (Invitrogen, Carlsbad, USA) with the small RNAs as the template. Investigated miRNA was amplified using forward primers that were designed based on mature miRNA sequences (Table 1) and reverse primers provided with the kit. The qRT-PCR amplification protocol was as follows: initial activation at 94°C for 2 min, followed by 40 cycles consisting of 94°C for 20 s and 60°C for 34 s. To verify the absence of contamination, negative controls (no cDNA template) were carried out. The experiments were performed for five technique replicates and three biological replicates. The data were calculated using the 2^−ΔΔ*C*T^ method [21]. 5.8S rRNA cloned from *A. crassicarpa* was selected as the reference gene for normalization.

**Table 1:** Forward primer sequences used for qRT-PCR.

Number	miRNA name	Primer sequence (5′-3′)
1	acr-miR159a-3p	GCTTTGGATTGAAGGGAGCTCTAA
2	acr-miR162	GCTCGATAAACCTCTGCATCCAAA
3	acr-miR390a-5p	CTCAGGAGGGATAGCGCCA
4	acr-miR396	CCTTCCACAGCTTTCTTGAACTGA
5	acr-miR319a	GCTTGGACTGAAGGGAGCTC
6	acr-miR156r	GCGGCCTTGACAGAAGATAGAGAGCATAAA
7	acr-miR164a	GGAGAAGCAGGGCACGTGCA
8	acr-miR166h-3p	GCTCTCGGACCAGGCTTCATTCC
9	acr-miR167a	GCGTGAAGCTGCCAGCATGATCTAAAA
10	acr-miR168a	CGCTTGGTGCAGGTCGGGAA
11	acr-miR171q-3p	GCGTGATTGAGCCGTGCCAATATCA
12	acr-miR397a-5p	GGGTCATTGAGTGCAGCGTTGATGAAA
13	acr-miR398d	GCTGTGTTCTCAGGTCGCCCC
14	acr-novel2*	GTCTTTCCAATGCCTCCCATACC
15	5.8S rRNA	CGCCTGCCTGGGTGTCACAA

### Statistical Analysis

The statistical analysis was performed using one-way Between-groups Linkage with SPSS 18.0 (SPSS Taiwan Corp., Taiwan).

## Results

### Small RNA Sequence Profile

**Figure 2:**
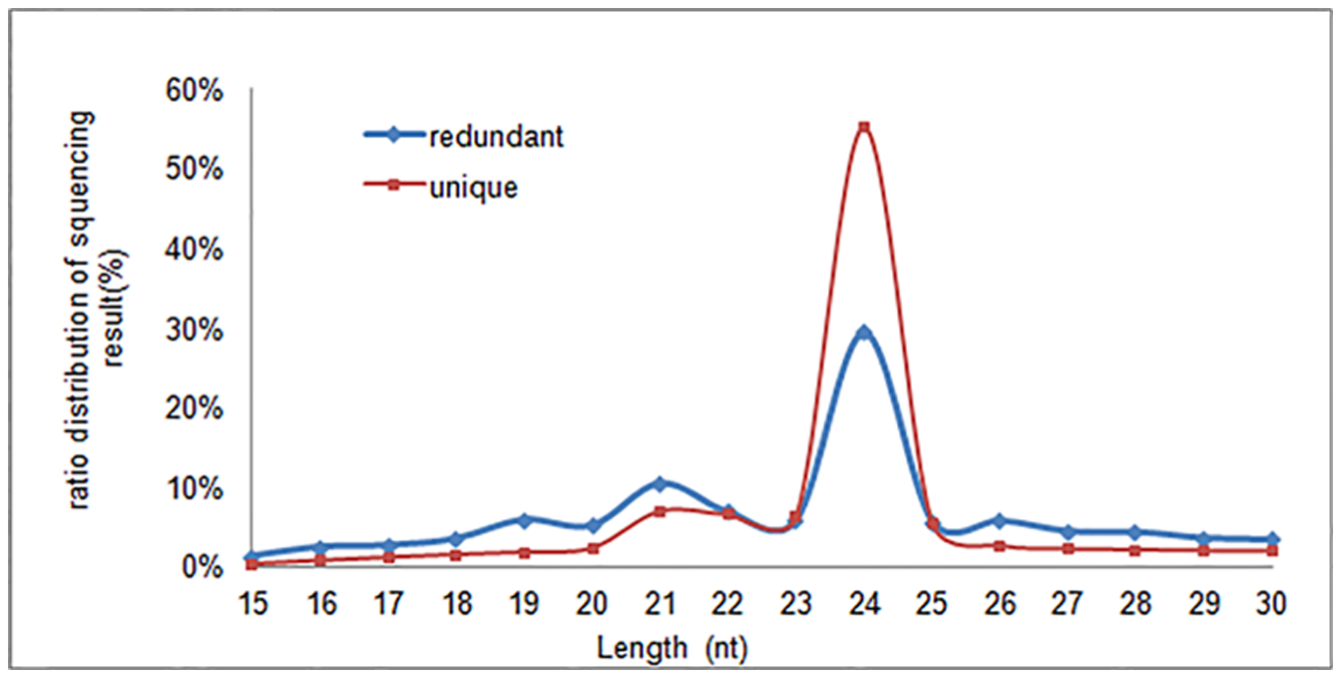
Length distribution of sequenced small RNAs. ‘Redundant’ represents the total number of sequences, ‘unique’ represents the number of unique sequences. nt, nucleotides.

A small RNA library was constructed using pooled RNAs isolated at six developmental stages of adventitious shoot regeneration, and sequenced using Solexa high-throughput technology in a single lane. More than 10 million raw reads were yielded, which were filtered for adaptors and junk contaminants. After removal of redundant sequences we obtained 980,584 unique sequences ranging from 15 to 30 nt in length, of which the majority was 19–26 nt long. A major peak in the abundance of mapped small RNAs was observed at 24 nt, which accounted for 55.3% of the sequences (Figure 2). Those sequences were perfectly mapped to pre-miRNAs of miRBase 19.0 and selected species genomes including *Arabidopsis thaliana*, *Populus trichocarpa* and soybean or *Acacia mangium* ESTs. The generated sequence was deemed a “mappable sequence”. The unmappable reads were further searched against noncoding RNAs (rRNA, tRNA, snRNA, and snoRNA) deposited in the Rfam and Repbase databases and comprised only a small fraction (4.59%) of the counts of all sequences, and were removed to avoid influencing miRNA identification (Table 2). Finally, 2,602,396 cleaned sequences representing 729,967 unique reads were used for subsequent analysis.

**Table 2:** Summary of reads from raw data to cleaned sequences of *Acacia crassicarpa.*

Category	Total reads		Unique reads	
raw reads	10,378,615	100%	980,584	100%
filtered reads	7,315,987	70.49%	203,692	20.78%
Rfam	449,801	4.33%	44,866	4.58%
Repeats	26,614	0.26%	4,116	0.42%
clean reads	2,602,396	25.07%	729,967	74.44%

Note: filtered reads: 5′ adapter, 3′ adapter, 5′ adapter and 3′ adapter joined together without insertion length with <15 nt and >32 nt and junk reads.

### Conserved miRNA Families and Isoforms

To identify conserved miRNAs in *A. crassicarpa*, we selected the 18–25 nt mappable small RNAs for further analysis by observation of miRNA length in miRBase. From a series of BLAST searches against miRBase 19.0 and *Arabidopsis thaliana*, *Populus trichocarpa* and soybean genomes or *Acacia mangium* or ESTs, we obtained 189 conserved unique sequences from 57 miRNA families allowing for sequence mismatches of not more than two nucleotides (Table 3). Among these sequences, the bulk of the miRNA families were conserved as we found orthologues of known miRNAs from all other plant species, such as *miR156*, *miR166*, *miR167* and *miR396*, whereas some miRNA families were less conserved and were present only in some of the plant species, such as *miR170* (conserved in *Arabidopsis* only), *miR6478* and *miR1448* (weakly conserved in *Populus trichocarpa*). Eight miRNA families were species-specific to the leguminous model plant soybean or *Medicago truncatula*, comprising *miR2118*, *miR4415*, *miR5368*, *miR4995*, *miR5281*, *miR2592*, *miR5740*, and *miR5256*.

**Table 3:** Identification of conserved miRNA families in *Acacia crassicarpa* with corresponding isoforms.

miRNA family	Gene name	Length	Sequence	Counts	Reference miRNA
MIR156	acr-miR156a	20	UGACAGAAGAGAGAGAGCAC	1	mtr-miR156a
	acr-miR156b	20	UGACAGAAGAGAGUGAGCAU	9	cca-miR156b
	acr-miR156c	23	UGACAGAAGAGAGUGAGCACCCA	1	ghr-miR156c
	acr-miR156d	20	UGACAGAAGAGAGUGAGCAC	584	gma-miR156a
	acr-miR156e	21	UUGACAGAAGAGAGCGAGCAC	80	sbi-miR156e
	acr-miR156e-p3	21	GCUCUCUAUUCUUCUGUCAUC	44	tcc-MIR156e-p3
	acr-miR156f	21	UGACAGAAGAGAGUGAGCACC	2	nta-miR156a
	acr-miR156g	18	ACAGAAGAGAGUGAGCAC	5	ath-miR156g
	acr-miR156h-5p	21	UUGACAGAAGACAGAGAGCAC	1	aly-miR156h-5p
	acr-miR156i-3p	22	GCUCACUUCUCUUUCUGUCAUC	5	mtr-MIR156i-p3
	acr-miR156j	21	UUGACAGAAGAGGGUGAGCAC	1	mtr-miR156j
	acr-miR156j-3p	22	GCUCACUUCUCUUUCUGUCAUU	2	cme-MIR156j-p3
	acr-miR156k	21	UUGACAGAAGAGAGAGAGCAC	17	gma-miR156b
	acr-miR156l	21	UUGACAGAAGAUGGAGAGCAC	3	ptc-miR156l
	acr-miR156m	21	UUGACAGAAGAUAGAGAGCAC	3530	gma-miR156c
	acr-miR156n	21	UUGACAGAAGAUAGAGGGCAC	7	mtr-miR156g
	acr-miR156o-3p	22	GCUCACUACUCUUUCUGUCAGU	1	gma-MIR156o-p3
	acr-miR156p	21	UUGACAGAAGAUAGGGAGCAC	4	gma-miR156p
	acr-miR156r	21	UUGACAGAAGAUAGAGAGCAU	58	gma-miR156r
	acr-miR157d-3p	21	GCUCUCUAUGCUUCUGUCAUC	9	ath-MIR157d-p3
	acr-miR157d-5p	20	UGACAGAAGAUAGAGAGCAC	27	ath-miR157d
MIR159	acr-miR159a-5p	19	GAGCUCCUUGAAGUCCAAU	2	gma-miR159a-5p
	acr-miR159a-3p	21	UUUGGAUUGAAGGGAGCUCUA	15030	gma-miR159a-3p
	acr-miR159b	21	GAGCUCCUUGAAGUCCAAUAG	18	ptc-MIR159b-p5
	acr-miR159c	19	UUUGGAUUGAAGGGAGCUC	127	ath-miR159c
	acr-miR159d	18	UUGGAUUGAAGGGAGCUC	8	ptc-miR159d
	acr-miR159e	20	UUUGGAUUGAAGGGAGCUCU	195	ath-miR159b
MIR160	acr-miR160a-5p	21	UGCCUGGCUCCCUGUAUGCCA	133	gma-miR160a-5p
	acr-miR160a-3p	21	GCGUAUGAGGAGCCAAGCAUA	7	gma-miR160a-3p
	acr-miR160b	21	CGUGGAUGGCGUAUGAGGAGC	1	ath-MIR160a-p3
MIR162	acr-miR162	21	UCGAUAAACCUCUGCAUCCAA	234	aau-miR162
	acr-MIR162-5p	21	GGAGGCAGCGGUUCAUCGAUC	3	aau-MIR162-p5
MIR164	acr-miR164a	21	UGGAGAAGCAGGGCACGUGCA	49	gma-miR164a
	acr-miR164c-5p	21	UGGAGAAGCAGGGCACGUGCC	4	aly-miR164c-5p
MIR165	acr-miR165a-3p	21	UCGGACCAGGCUUCAUCCCCC	7	aly-miR165a-3p
	acr-miR165a-5p	23	GGAAUGUUGUCUGGCUCGAGGAU	1	aly-miR165a-5p
MIR166	acr-miR166a	21	UCGAACCAGGCUUCAUUCCCC	8	rco-miR166a
	acr-miR166a-3p	21	UCGGACCAGGCUUCAUUCCCC	9042	gma-miR166a-3p
	acr-miR166a-5p	21	GGAAUGUUGUCUGGCUCGAGG	137	gma-miR166a-5p
	acr-miR166b-5p	21	GGAAUGUUGUCUGGUUCGAGG	1	osa-miR166b-5p
	acr-miR166c-5p	20	GGAAUGUCGUCUGGUUCGAU	1	ptc-MIR166e-p5
	acr-miR166d-3p	21	UCGGGCCAGGCUUCAUUCCCC	3	mtr-miR166d
	acr-miR166d-5p	21	GGAAUGUUGCCUGGCUCGAGG	1	mtr-MIR166d-p5
	acr-miR166e-5p	21	GGAAUGUUGGCUGGCUCGAGG	16	gma-MIR166e-p5
	acr-miR166f-3p	20	UCUCGGACCAGGCUUCAUCC	1	bna-MIR166f-p3
	acr-miR166g-5p	21	GGAAUGCUGUCUGGUUCGAGA	1	ptc-MIR166g-p5
	acr-miR166h-3p	21	UCUCGGACCAGGCUUCAUUCC	2271	gma-miR166h-3p
	acr-miR166i	23	UCGGACCAGGCUUCAUUCCCCUU	3	cme-miR166i
	acr-miR166i-5p	21	GGAAUGUCGUCUGGUUCGAGA	11	gma-miR166i-5p
	acr-miR166j	21	UCUCGGACCAGGCUCCAUUCC	1	ptc-miR166p
	acr-miR166l-3p	21	UCGGACCAGGCUUCAUUUCCC	10	gma-MIR166l-p3
	acr-miR166m	19	GGACCAGGCUUCAUUCCCC	25	gma-miR166m
	acr-miR166p	19	UCGGACCAGGCUUCAUUCC	34	gma-miR166p
MIR167	acr-miR167a	21	UGAAGCUGCCAGCAUGAUCUA	64	gma-miR167a
	acr-miR167a-3p	23	GGUCAUGCUGUGACAGCCUCACU	5	gma-MIR167a-p3
	acr-miR167b-3p	21	AGAUCAUGCGGCAGUUUCACC	6	csi-MIR167b-p3
	acr-miR167b-5p	23	UGAAGCUGCCAGCAUGAUCUGGG	3	mtr-miR167b-5p
	acr-miR167c	21	UGAAGCUGCCAGCAUGAUCUC	3	vvi-miR167c
	acr-miR167d	22	UGAAGCUGCCAGCAUGAUCUGA	266	gma-miR167c
	acr-miR167e	22	UGAAGCUGCCAGCAUGAUCUUA	9	gma-miR167e
	acr-miR167e-3p	20	GAUCAUGUGGCAGUUUCACC	4	gma-MIR167e-p3
	acr-miR167f	23	UGAAGCUGCCAGCAUGAUCUGAC	4	dpr-miR167c
	acr-miR167g	22	UGAAGCUGCCAGCAUGAUCUGG	137	gma-miR167c
MIR168	acr-miR168a	21	UCGCUUGGUGCAGGUCGGGAA	456	gma-miR168a
	acr-miR168a-3p	21	CCCGCCUUGCAUCAACUGAAU	322	gma-MIR168a-p3
	acr-miR168b	21	UUGCUUGGUGCAGGUCGGGAA	2	mtr-miR168a
	acr-miR168b-3p	22	CCCGCCUUGCAUCAACUGAAUU	8	nta-MIR168a-p3
MIR169	acr-miR169a	21	CAGCCAAGGAUGACUUGCCGG	5	gma-miR169a
	acr-miR169a-5p	21	AUGCAGCCAAGGAUGACUUGC	1	ptc-MIR169a-p5
	acr-miR169a-3p	23	GGCAAGUUGUCCUUGGCUACACU	1	ath-MIR169a-p3
	acr-miR169aa	21	CAGCCAAGAAUGACUUGUCGG	1	ptc-miR169aa
	acr-miR169d-5p	20	AGCCAAGGAUGACUUGCCGG	2	aly-miR169d-5p
	acr-miR169b-3p	21	GGCAAGUUGUCUUUGGCUAUG	3	gma-MIR169a-p3
	acr-miR169e	24	UGAGCCAAGGAUGACUUGCCGGCC	5	gma-miR169e
	acr-miR169j-5p	21	AGCCAAGAAUGACUUGCCGGA	4	gma-miR169j-5p
	acr-miR169k	21	CAGCCAAGAAUGACUUGCCGG	49	gma-miR169k
	acr-miR169m	24	UGAGCCAAGGAUGACUUGCCGGCA	16	vvi-miR169m
	acr-miR169p	19	UGAGCCAAGGAUGACUUGC	1	gma-miR169p
	acr-miR169r	22	UGAGCCAAGAAUGACUUGCCGG	16	cme-miR169r
	acr-miR169z	22	CAGCCAAGAAUGACUUGCCGGC	2	ptc-miR169z
MIR171	acr-miR171a	21	UUGAGCCGUGCCAAUAUCACG	7	gma-miR171a
	acr-miR171c-5p	21	AGAUAUUGGUGCGGUUCAAUA	1	aly-miR171c-5p
	acr-miR171b-3p	21	UGAGCCGAAUCAAUAUCACUC	9	gma-miR171b-3p
	acr-miR171e-5p	22	UAUUGGCCUGGUUCACUCAGAA	3	mtr-MIR171c-p5
	acr-miR171d-3p	22	UUGAGCCGUGCCAAUAUCACAA	1	zma-miR171b-3p
	acr-miR171f-5p	21	AGAUAUUGGUACGGUUCAAUC	5	gma-MIR171f-p5
	acr-miR171j-5p	21	UAUUGGCCUGGUUCACUCAGA	12	gma-miR171j-5p
	acr-miR171k-3p	21	UUGAGCCGCGCCAAUAUCACU	8	gma-miR171k-3p
	acr-miR171m	21	UUGAGCCGCGUCAAUAUCUCA	1	gma-miR171m
	acr-miR171q-3p	21	UGAUUGAGCCGUGCCAAUAUC	11	gma-MIR171q-p3
MIR172	acr-miR172a-3p	21	AGAAUCUUGAUGAUGCUGCAU	3	aly-miR172a-3p
	acr-miR172c	21	GGAAUCUUGAUGAUGCUGCAC	49	gma-miR172c
	acr-miR172d-5p	21	GCGGCAUCAUUAAGAUUCACA	7	ptc-MIR172d-p5
	acr-miR172c-3p	21	AGAAUCUUGAUGAUGCUGCAG	45	aly-miR172c-3p
	acr-miR172e	22	AGAAUCUUGAUGAUGCUGCAGU	7	cme-miR172e
MIR2111	acr-miR2111a	21	UAAUCUGCAUCCUGAGGUUUU	1	ptc-miR2111a
	acr-miR2111a-5p	21	UAAUCUGCAUCCUGAGGUAUA	8	aly-miR2111a-5p
	acr-miR2111b	21	AAUCUGCAUCCUGAGGUAUAG	26	gma-miR2111b
MIR2118	acr-miR2118a-3p	22	UUUCCGAUUCCACCCAUUCCUA	912	gma-miR2118a-3p
MIR2590	acr-miR2590d-5p	18	UCAGCGUGGUCGGAAAUC	1	mtr-MIR2590d-p5
MIR2592	acr-miR2592bj-5p	18	AUUCCCACUGUCCCUGUC	3	mtr-MIR2592bj-p5
MIR2631	acr-miR2631-3p	19	UUCAAUUGUAAAAUUUUGU	2	mtr-MIR2631-p3
MIR2655	acr-miR2655n-5p	21	UUUGAUCCUUUCUGUAAAUUU	2	mtr-MIR2655n-p5
	acr-miR2655o-3p	21	UAAAUUUACAGAAAGGAUCAA	2	mtr-MIR2655o-p3
MIR2911	acr-miR2911	23	GCCGGCCGGGGGACGGACUGGGA	314	han-miR2911
	acr-miR2911-5p	23	CCCGAACCCGUCGGCUGUCGGCG	500	han-MIR2911-p5
MIR2916	acr-miR2916-5p	24	CUAGUCUCAACCAUAAACGAUGCC	42	peu-MIR2916-p5
	acr-miR2916-3p	18	GCGGAUGUUGCUUUUAGG	31	peu-MIR2916-p3
MIR319	acr-miR319	25	UUUGGACUGAAGGGAGCUCCUAAUU	2	tcc-miR319
	acr-miR319a	20	UUGGACUGAAGGGAGCUCCC	3259	gma-miR319a
	acr-miR319f	19	UUGGACUGAAGGGAGCUCC	43	gma-miR319g
	acr-miR319g	21	UUUGGACUGAAGGGAGCUCCU	689	gma-miR319g
	acr-miR319h-5p	21	AGCUGCUGACUCAUUCAUUCA	12	gma-MIR319h-p5
	acr-miR319i	21	UUUGGACUGAAGGGAGCUCCC	17	ptc-miR319i
	acr-miR319n	21	UUUGGACCGAAGGGAGCUCCU	1	gma-miR319n
MIR390	acr-miR390a	22	AAGCUCAGGAGGGAUAGCGCCU	3	cme-miR390a
	acr-miR390a-3p	21	CGCUAUCCAUCCUGAGUUUCA	1	gma-miR390a-3p
	acr-miR390a-5p	21	AAGCUCAGGAGGGAUAGCGCC	398	gma-miR390a-5p
	acr-miR390g-3p	21	CGCUAUCUAUCCUGAGUUUUA	3	gma-MIR390g-p3
MIR393	acr-miR393a	22	UCCAAAGGGAUCGCAUUGAUCU	9	gma-miR393a
	acr-miR393b-3p	21	UAGGAUCAUGCUAUCCCUUUG	1	gma-MIR393b-p3
	acr-miR393i-3p	21	AUCAUGCGAUCUCUUAGGAAU	2	gma-MIR393i-p3
MIR394	acr-miR394a-5p	20	UUGGCAUUCUGUCCACCUCC	69	gma-miR394a-5p
MIR395	acr-miR395a	21	UGAAGUGUUUGGGGGAACUCC	104	gma-miR395a
	acr-miR395a-5p	18	GUUCCCCUGAACACUUCA	2	gma-MIR395a-p5
	acr-miR395b	21	CUGAAGUGUUUGGAGGAACUC	2	cca-miR395b
	acr-miR395c	21	CUGAAGUGUUUGGGGGAACUC	24	ptc-miR395b
	acr-miR395d	21	CUGAAGUGUUUGGGGGAACUU	1	gma-miR395d
	acr-miR395g-5p	21	AGUUCCUCUGAACACUUCACC	63	gma-MIR395g-p5
	acr-miR395h-5p	21	AGUUCCUCUGAACGCUUCAUU	1	gma-MIR395h-p5
	acr-miR395i	20	UGAAGUGUUUGGGGGAACUC	8	gma-miR395i
MIR396	acr-miR396	22	UUUCCACAGCUUUCUUGAACUG	7	dpr-miR396
	acr-miR396c	21	UUCCACAGCUUUCUUGAACUG	13334	aau-miR396
	acr-miR396-3p	21	GUUCAAUAAAGCUGUGGGAAG	167	aau-MIR396-p3
	acr-miR396a-5p	22	AUUCCACAGCUUUCUUGAACUG	56	gma-miR396a-5p
	acr-miR396a-3p	21	AUUCAAGAAAGCUGUGGAAAA	13	csi-MIR396a-p3
	acr-miR396b-5p	21	UUCCACAGCUUUCUUGAACUU	261	gma-miR396b-5p
	acr-miR396b-3p	21	GCUCAAGAAAGCUGUGGGAUA	29	gma-miR396b-3p
	acr-miR396e	22	UUCCACGGCUUUCUUGAACUGC	32	cme-miR396e
	acr-miR396f	21	UUCCACGGCUUUCUUGAACUG	1388	ptc-miR396f
	acr-miR396g-5p	19	UUCCACGGCUUUCUUGAAC	2	ptc-miR396g-5p
	acr-miR396j-5p	21	UUCCACAGUUUUCUUGAACUG	3	gma-MIR396j-p5
MIR397	acr-miR397a	21	AUUGAGUGCAGCGUUGAUGAA	28	gma-miR397a
	acr-miR397a-5p	21	UCAUUGAGUGCAGCGUUGAUG	12	aly-miR397a-5p
	acr-miR397b-3p	21	AUCGACGCUGCACUCAAUCAU	43	gma-miR397b-3p
	acr-miR397c	21	CAUUGAGUGCAGCUUUGAUGA	1	ptc-miR397c
MIR398	acr-miR398	22	UGUGUUCUCAGGUCGCCCCUGU	3	nta-miR398
	acr-miR398a	23	UGUGUUCUCAGGUCGCCCCUGUU	1	bdi-miR398a
	acr-miR398d	21	UGUGUUCUCAGGUCGCCCCUU	4	gma-miR398a
	acr-miR398b	21	UGUGUUCUCAGGUCCCCCCUG	1	ath-miR398b
	acr-miR398c	21	UGUGUUCUCAGGUCGCCCCUG	181	gma-miR398c
MIR399	acr-miR399a	20	UGCCAAAGGAGAGUUGCCCU	1	gma-miR399a
	acr-miR399b	21	UGCCAAAGGAGAAUUGCCCUG	2	vun-miR399b
	acr-miR399d	21	UGCCAAAGGAGAGUUGCCCUU	8	cme-miR399d
MIR403	acr-miR403-3p	21	UUAGAUUCACGCACAAACUCG	975	aly-miR403-3p
	acr-miR403a	19	UUAGAUUCACGCACAAACU	3	gma-miR403a
MIR408	acr-miR408-5p	21	CUGGGAACAGGCAGAGCAUGG	44	ptc-miR408-5p
	acr-miR408a-3p	21	AUGCACUGCCUCUUCCCUGGC	157	gma-miR408a-3p
	acr-miR408d-5p	21	GCUGGGAACAGGCAGAGCAUG	19	gma-MIR408d-p5
MIR4414	acr-miR4414b	21	UGUGAAUGAUGCAGGAGCUAA	5	mtr-miR4414b
MIR4415	acr-miR4415a-3p	21	UUGAUUCUCAUCACAACAUGC	305	gma-miR4415a-3p
MIR4416	acr-miR4416c-3p	21	AUACGGGUCGCUCUCACCUGG	2	gma-MIR4416c-p3
MIR482	acr-miR482-5p	21	GGAAUGGGCUGUUUGGGAAGC	1	pvu-miR482-5p
	acr-miR482a-5p	20	GGAAUGGGCUGUUUGGGAAG	7	mdm-miR482a-5p
MIR4995	acr-miR4995-5p	20	UGGCUUGGUUAAGGGAACCC	5	gma-MIR4995-p5
MIR5015	acr-miR5015-5p	24	UUUUGUUGUUGUUGUUAUUAUGUU	1	ath-MIR5015-p5
MIR5030	acr-miR5030-3p	20	UUCCUGAAGAACAAAUUGUU	1	gma-MIR5030-p3
MIR5139	acr-miR5139	18	AACCUGGCUCUGAUACCA	282	rgl-miR5139
MIR5248	acr-miR5248	21	UUUUUAGUUGGCAUGCAUUUA	2	mtr-miR5248
MIR5256	acr-miR5256-5p	21	UUGUAAGAUUAAAAUGGUUGA	3	mtr-MIR5256-p5
MIR5257	acr-miR5257-5p	21	UCAUCAAGAACAAGUAGAACU	1	mtr-MIR5257-p5
MIR5261	acr-miR5261	21	UUCAUUGUAAAUGGCUUUGGC	1	mtr-miR5261
MIR5281	acr-miR5281b	24	CUUAUAAUUAGGACCGGAGGGAGU	6	mtr-miR5281b
	acr-miR5281c-5p	23	UAUAUAUUACUACCUUCGGUCCU	1	mtr-MIR5281c-p5
MIR5282	acr-miR5282-3p	24	GCAAAAUUUUGUGACGGAAUUAGU	1	mtr-MIR5282-p3
MIR530	acr-miR530a	22	UGCAUUUGCACCUGCACUUUAA	1	gma-miR530a
MIR5368	acr-miR5368-3p	19	GAUACCACUCUGGAAGAGC	96	gma-MIR5368-p3
	acr-miR5368-5p	20	CCUGGGAUUGGCUUUGGGCC	16	gma-MIR5368-p5
MIR5740	acr-miR5740-5p	21	UUGAUUUGUAUCUGUUUGGAU	3	mtr-MIR5740-p5
MIR6478	acr-miR6478	25	CCGACCUUAGCUCAGUUGGUAGAGC	190	ptc-miR6478
MIR6485	acr-miR6485-5p	23	UCGGCAGAUUUGGAUUCCUAUAU	3	hbr-MIR6485-p5
MIR827	acr-miR827	18	UUAGAUGGCCAUCAACGA	1	pUc-miR827
MIR828	acr-miR828	22	ACUUGCUCAAAUGAGUAUUCCA	1	gma-miR828a
MIR858	acr-miR858a	21	UUUCGUUGUCUGUUCGACCUU	12	ath-miR858a
	acr-miR858b	21	UUCGUUGUCUGUUCGACCUUG	7	ath-miR858b
MIR869	acr-miR869-5p	18	CAACUCCAGGAUUGAACC	1	ath-MIR869-p5
MIR1310	acr-miR1310-3p	20	AAACUUUAAAUAGGUAGGAC	66	han-MIR1310-p3
MIR1448	acr-miR1448-3p	20	UUUCCAAUGCCUCCCAUACC	14	ptc-MIR1448-p3
MIR1511	acr-miR1511	18	AACCAGGCUCUGAUACCA	11	gma-miR1511

The majority of conserved miRNA families contained members that differed in no more than two nucleotides, which are regarded as miRNA isoforms [22]. Interestingly, the number of isoforms in differently conserved miRNA families varied considerably. Four families (*miR156*, *miR166*, *miR167*, and *miR396*) contained the highest number of multiple isoforms with 13, 11, 10, and 10 members, respectively. Eighteen miRNA families (e.g., *miR165*, *miR170*, *miR393*, *miR399*, and *miR1511*) contained only one member.

The abundance of known miRNA families was also monitored. Interestingly, a notable divergence in expression frequency among miRNAs during shoot regeneration was observed. For example, *miR159*, *miR166*, and *miR396* were detected most frequently with the counts of 15,425, 11,560, and 15,290, respectively; *miR156*, *miR157*, and *miR319* showed moderate abundance with counts of 4355, 3566, and 4020, respectively; and miRNAs such as *miR2592*, *miR5256*, *miR5740*, and *miR6485* showed low abundance (Table 3).

### Putative Novel miRNAs

**Figure 3:**
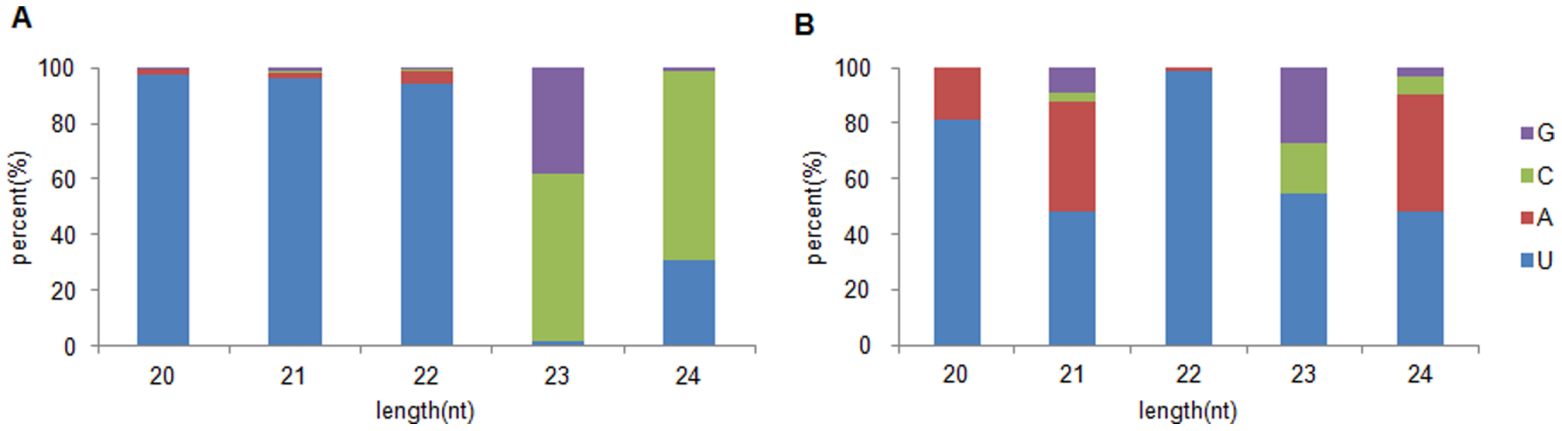
First nucleotide bias of identified miRNAs in *Acacia crassicarpa*. (A) Conserved miRNAs, (B) novel miRNA candidates.

**Figure 4:**
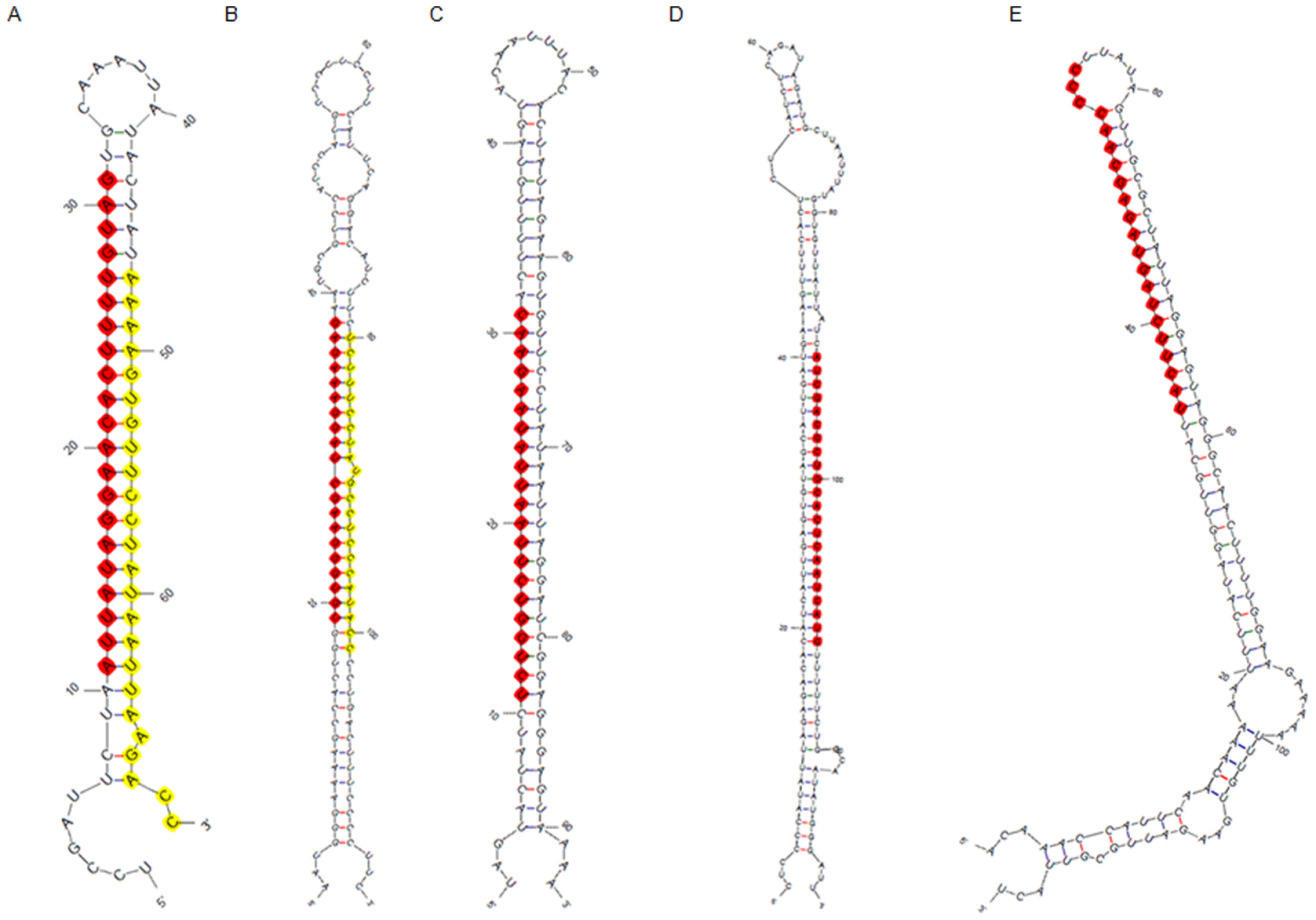
Stem-loop structure of partial novel miRNA precursors. (A) acr-novel1 and acr-novel1*, (B) acr-novel2 and acr-novel2*, (C) acr-novel3, (D) acr-novel4,(E) acr-novel5. Novel miRNA sequences were noted by red and yellow shadow.

The sequences unmapped to the pre-miRNAs in miRBase 19.0 were chosen to identify putative novel miRNAs. The following criteria were applied: (1) the unique sequences were mapped to the selected species genomes; (2) the extended sequence at the mapped genome location had the propensity to form a hairpin. Taken together, a total of 7 novel miRNA candidates from 5 families were obtained with a minimal folding free energy index (MFEI) for the hairpin structure of the miRNA precursor of less than 0.7 (Table 4). These miRNA candidates commonly displayed a strong bias toward a 5′ U at the first nucleotide position (Figure 3) and stem-loop structure precursors (Figure 4), which was consistent with conserved miRNAs [23]. Some previous studies reported that a standard for discrimination between high-confidence microRNAs and fragments of other RNAs in deep sequencing data was the detection of miRNA* sequences [23]. Among the miRNA candidates in the present study, four novel miRNAs were identified with their complementary miRNA* belonging to two miRNA families, which implied that these putative miRNAs were most likely novel to *A. crassicarpa*, and several potentially novel miRNAs might be specific to *A. crassicarpa* or Fabaceae species. Of these potentially novel miRNAs, the copy number of several miRNA*s was low. This feature is consistent with miRNA* degradation during the miRNA generation procedure [24], so counts of miRNA*s were not included in the most recent criteria for annotation of plant miRNAs. In the present study, acr-novel2* showed higher counts than the corresponding novel miRNAs. This finding was in accordance with the recent discovery by Zhang *et al.* [25] that miRNA* sequences are abundantly expressed.

**Table 4:** Novel miRNA families identified from *Acacia crassicarpa.*

miRNA	Location	Sequence (5′-3′)	Length	Count	MFEI
acr-novel1	gi|293578058:1:181−	AUUAUAGGAACACUUUUGUAG	21	22	1.6
acr-novel1*		AAAAGUGUUCCUAUAAUUAGGACC	24	2	1.6
acr-novel2	gi|293579709:1:247+	UGUGGGACGCUAGGAAAGAG	20	2	0.9
acr-novel2*		UCUUUCCUAUGCCUCCCAUACC	22	4515	0.9
acr-novel3	gi|293581175:360:620−	UCCGGUCUUAAUUAUAAGAAC	21	7	1.6
acr-novel4	gma_Gm07:35780857:35781118+	AUCGACGCUGCACUCAAUCAUU	22	58	1
acr-novel5	ptr_Chr13:8496156:8496416−	UACUUCAAGUAGAGCAACCCC	21	7	0.9

### Putative Target Prediction for Conserved and Novel miRNAs

Putative targets of identified conserved and novel miRNAs were predicted by BLAST using mRNA sequences from *A. thaliana* [22] using TargetFinder. On the basis of completely complementarity between the miRNA and target mRNA [26], we screened 237 genes for feasible targets with a total score not higher than 3 (Table 5). Among the targets predicted, the most frequent were transcription factors, including a SBP transcription factor, MYB transcription factor, NAC transcription factor and GRF transcription factor, and other putative target genes involved in a range of physiological or metabolic processes, for example functional proteins such as protein kinases, laccase, and protein phosphatase. Many reports have demonstrated that target genes of conserved miRNAs are consistent among the majority of plant species [27], and this was the case in the present study. Putative target genes of *miR156/157* mainly encoded a SBP transcription factor family protein and SQUAMOSA promoter binding protein (*SPL*), which are involved in leaf and flower development. The *miR160* target mRNA was annotated with an auxin response factor (*ARF*), which might be associated with auxin response during plant organogenesis [28]. These results provide valuable information for the further research.

**Table 5:** Predicted target genes of miRNAs in *Acacia crassicarpa.*

miRNA	Target	Score	Annotation
MIR156	AT1G69170	1	SBP domain transcription factor family protein
	AT5G50570	1	SBP domain transcription factor family protein
	AT5G50670	1	SBP domain transcription factor family protein
	AT1G53160	2	squamosa promoter binding protein-like 4
	AT1G71690	3	Protein of unknown function (DUF579)
	AT3G15270	3	squamosa promoter binding protein-like 5
	AT5G10945	3	MIR156D; miRNA
	AT5G10946	3	unknown protein
	AT2G19420	0	unknown protein
	AT1G29900	2	carbamoyl phosphate synthetase B
	AT2G33810	2	squamosa promoter binding protein-like 3
	AT3G28690	2	Protein kinase superfamily protein
	AT1G17500	3	ATPase E1-E2 type family protein/haloaciddehalogenase-like hydrolase family protein
	AT1G29030	3	Apoptosis inhibitory protein 5 (API5)
	AT1G44790	3	ChaC-like family protein
	AT1G48410	3	Stabilizer of iron transporter SufD/Polynucleotidyltransferase
	AT1G61580	3	R-protein L3 B
	AT1G62305	3	Core-2/I-branchingbeta-1,6-N-acetylglucosaminyltransferasefamily protein
	AT1G71240	3	Plant protein of unknown function (DUF639)
	AT2G11910	3	unknown protein
	AT3G04870	3	zeta-carotene desaturase
	AT3G06670	3	binding
	AT3G18210	3	2-oxoglutarate (2OG) and Fe(II)-dependentoxygenase superfamily protein
	AT5G32566	3	transposable element gene
	AT1G27360	0	squamosa promoter-like 11
	AT1G27370	0	squamosa promoter binding protein-like 10
	AT2G42200	0	squamosa promoter binding protein-like 9
	AT3G57920	0	squamosa promoter binding protein-like 15
	AT5G43270	0	squamosa promoter binding protein-like 2
	AT1G22000	3	FBD, F-box and Leucine Rich Repeatdomains containing protein
	AT3G44096	3	transposable element gene
	AT3G47170	3	HXXXD-type acyl-transferase family protein
	AT5G66600	3	Protein of unknown function, DUF547
	AT1G16916	3	unknown protein
	AT4G24270	3	EMBRYO DEFECTIVE 140
	AT5G11977	3	MIR156E; miRNA
	AT5G08620	3	DEA(D/H)-box RNA helicase family protein
	AT5G24930	3	CONSTANS-like 4
	AT5G44280	3	RING 1A
	AT5G55590	3	Pectin lyase-like superfamily protein
	AT5G55835	3	MIR156H; miRNA
	AT5G20380	3	phosphate transporter 4;5
	AT1G48742	2	MIR157D; miRNA
	AT3G18217	2	MIR157C; miRNA
	AT1G30450	3	cation-chloride co-transporter 1
	AT1G73650	3	Protein of unknown function (DUF1295)
	AT3G13490	3	Lysyl-tRNA synthetase, class II
	AT4G31050	3	Biotin/lipoate A/B protein ligase family
	AT5G35930	3	AMP-dependent synthetase and ligasefamily protein
	AT4G05400	3	copper ion binding
MIR159	AT2G34010	3	unknown protein
	AT4G37770	2	1-amino-cyclopropane-1-carboxylate synthase 8
	AT3G60460	3	myb-like HTH transcriptional regulatorfamily protein
	AT4G22415	3	transposable element gene
	AT3G28915	3	transposable element gene
	AT3G33133	3	transposable element gene
	AT5G65540	3	unknown protein
	AT2G26950	2	myb domain protein 104
	AT3G11440	2	myb domain protein 65
	AT3G52030	2	F-box family protein with WD40/YVTNrepeat doamin
	AT5G06100	2	myb domain protein 33
	AT1G08030	3	tyrosylprotein sulfotransferase
	AT1G52100	3	Mannose-binding lectin superfamily protein
	AT1G76390	3	ARM repeat superfamily protein
	AT2G32460	3	myb domain protein 101
	AT3G33076	3	transposable element gene
	AT3G33084	3	transposable element gene
	AT4G26930	3	myb domain protein 97
	AT4G27330	3	sporocyteless (SPL)
	AT5G16810	3	Protein kinase superfamily protein
	AT5G28335	3	transposable element gene
	AT5G55020	3	myb domain protein 120
MIR160	AT2G28350	2	auxin response factor 10
MIR164	AT1G56010	2	NAC domain containing protein 1
	AT3G15170	3	NAC domain transcriptional regulatorsuperfamily protein
	AT5G53950	3	NAC domain transcriptional regulatorsuperfamily protein
MIR167	AT3G04765	3	MIR167C; miRNA
	AT3G07120	3	RING/U-box superfamily protein
MIR169	AT3G05690	2	nuclear factor Y, subunit A2
	AT5G06510	2	nuclear factor Y, subunit A10
	AT1G68560	3	alpha-xylosidase 1
	AT2G46020	3	transcription regulatory protein SNF2,putative
	AT5G23110	3	Zinc finger, C3HC4 type (RING finger)family protein
	AT1G17590	3	nuclear factor Y, subunit A8
	AT1G54160	3	nuclear factor Y, subunit A5
	AT5G42120	3	Concanavalin A-like lectin protein kinasefamily protein
	AT5G12840	2	nuclear factor Y, subunit A1
	AT3G20910	3	nuclear factor Y, subunit A9
MIR171	AT2G45160	1	GRAS family transcription factor
	AT3G60630	1	GRAS family transcription factor
	AT4G00150	1	GRAS family transcription factor
	AT1G62035	3	MIR171C; miRNA
MIR172	AT5G60120	1	target of early activation tagged (EAT) 2
	AT5G65790	2	myb domain protein 68
	AT5G67180	2	target of early activation tagged (EAT) 3
	AT1G61290	3	syntaxin of plants 124
	AT3G11435	3	MIR172C; miRNA
	AT5G12900	3	unknown protein
	AT4G36920	1	Integrase-type DNA-binding superfamilyprotein
	AT2G28056	2	MIR172/MIR172A; miRNA
	AT2G28550	2	related to AP2.7
	AT2G39250	3	Integrase-type DNA-binding superfamilyprotein
	AT3G49690	3	myb domain protein 84
	AT3G54990	2	Integrase-type DNA-binding superfamilyprotein
	AT3G55512	3	MIR172D; miRNA
	AT5G04275	3	MIR172/MIR172B; miRNA
	AT5G59505	3	MIR172E; miRNA
MIR319	AT2G26950	2	myb domain protein 104
	AT3G11440	2	myb domain protein 65
	AT5G06100	2	myb domain protein 33
	AT1G53230	3	TEOSINTE BRANCHED 1, cycloidea andPCF transcription factor 3
	AT3G33076	3	transposable element gene
	AT3G33084	3	transposable element gene
	AT3G66658	3	aldehyde dehydrogenase 22A1
	AT4G22415	3	transposable element gene
MIR390	AT2G38325	3	MIR390A; miRNA
MIR393	AT3G62980	1	F-box/RNI-like superfamily protein
	AT4G03190	2	GRR1-like protein 1
MIR394	AT1G27340	1	Galactose oxidase/kelch repeat superfamilyprotein
	AT3G29660	3	transposable element gene
	AT5G09670	3	loricrin-related
	AT5G09672	3	conserved peptide upstream open reading frame 21
MIR395	AT1G26973	2	MIR395A; miRNA
	AT1G69792	2	MIR395D; miRNA
	AT1G69795	2	MIR395E; miRNA
	AT1G26975	3	MIR395B; miRNA
	AT1G26985	3	MIR395C; miRNA
	AT1G69797	3	MIR395F; miRNA
	AT2G41060	3	RNA-binding (RRM/RBD/RNP motifs)family protein
	AT3G32280	3	ATP-dependent helicase family protein
	AT4G14770	3	TESMIN/TSO1-like CXC 2
	AT5G04140	3	glutamate synthase 1
	AT5G19900	3	PRLI-interacting factor, putative
	AT2G28780	2	unknown protein
	AT1G50930	1	unknown protein
	AT5G43780	1	Pseudouridine synthase/archaeosinetransglycosylase-like family protein
	AT3G22890	3	ATP sulfurylase 1
	AT4G14680	3	Pseudouridine synthase/archaeosinetransglycosylase-like family protein
	AT4G06509	3	transposable element gene
	AT5G13630	3	magnesium-chelatase subunit chlH,chloroplast, putative/Mg-protoporphyrinIX chelatase
MIR396	AT5G14130	3	Peroxidase superfamily protein
	AT1G58020	2	transposable element gene
	AT2G45480	3	growth-regulating factor 9
	AT3G33106	3	transposable element gene
	AT5G01370	3	ALC-interacting protein 1
	AT5G07700	3	myb domain protein 76
	AT5G35407	1	MIR396B; miRNA
	AT2G36400	3	growth-regulating factor 3
	AT3G01910	3	sulfite oxidase
	AT2G04270	3	RNAse E/G-like
	AT3G52910	3	growth-regulating factor 4
	AT5G16690	3	origin recognition complex subunit 3
MIR397	AT5G18420	3	unknown protein
	AT2G29130	2	laccase 2
	AT4G05105	2	MIR397A; miRNA
MIR398	AT5G14550	1	Core-2/I-branchingbeta-1,6-N-acetylglucosaminyltransferase family protein
MIR403	AT1G31280	0	Argonaute family protein
	AT1G03060	3	Beige/BEACH domain;WD domain,G-beta repeat protein
	AT4G30620	3	Uncharacterised BCR, YbaB familyCOG0718
MIR408	AT2G47020	0	Peptide chain release factor 1
	AT2G02850	1	plantacyanin
MIR857	AT1G14610	2	valyl-tRNA synthetase/valine–tRNAligase (VALRS)
	AT1G74060	2	Ribosomal protein L6 family protein
	AT5G28350	2	Quinoprotein amine dehydrogenase
	AT1G06670	3	nuclear DEIH-boxhelicase
	AT1G17040	3	SH2 domain protein A
	AT1G33475	3	SNARE-like superfamily protein
	AT1G43590	3	transposable element gene
	AT1G48010	3	Plant invertase/pectin methylesteraseinhibitor superfamily protein
	AT3G20090	3	cytochrome P450, family 705, subfamily A,polypeptide 18
	AT3G54230	3	suppressor of abi3-5
	AT3G57660	3	nuclear RNA polymerase A1
	AT3G61480	3	Quinoprotein amine dehydrogenase, betachain-like; RIC1-like guanyl-nucleotideexchange factor
	AT4G08460	3	Protein of unknown function (DUF1644)
	AT4G10170	3	SNARE-like superfamily protein
	AT5G06040	3	self-incompatibility protein-related
	AT5G59470	3	Mannose-P-dolichol utilization defect 1 protein
MIR858	AT1G06180	3	myb domain protein 13
	AT1G66230	3	myb domain protein 20
	AT2G47460	3	myb domain protein 12
	AT3G08500	3	myb domain protein 83
	AT4G12350	3	myb domain protein 42
	AT5G35550	3	Duplicated homeodomain-likesuperfamily protein
	AT5G49330	3	myb domain protein 111
MIR1310	AT2G16592	3	Bifunctional inhibitor/lipid-transfer protein/seedstorage 2S albumin superfamily protein
	AT3G29050	3	receptor-like protein kinase-related
MIR1448	AT5G40100	1	Disease resistance protein (TIR-NBS-LRR class)family
	AT1G65790	2	receptor kinase 1
	AT1G65800	2	receptor kinase 2
	AT3G46530	2	NB-ARC domain-containing diseaseresistance protein
	AT1G63350	3	Disease resistance protein (CC-NBS-LRR class)family
	AT2G30740	3	Protein kinase superfamily protein
	AT4G00420	3	Double-stranded RNA-binding domain(DsRBD)-containing protein
MIR1511	AT3G28160	2	transposable element gene
	AT5G42820	2	Zinc finger C-x8-C-x5-C-x3-H type family protein
	AT1G03050	3	ENTH/ANTH/VHS superfamily protein
	AT1G17340	3	Phosphoinositide phosphatase family protein
	AT1G61610	3	S-locus lectin protein kinase family protein
	AT3G21360	3	2-oxoglutarate (2OG) and Fe(II)-dependentoxygenase superfamily protein
	AT4G24450	3	phosphoglucan, water dikinase
	AT5G27965	3	transposable element gene
	AT5G32690	3	Pseudogene of AT2G29880
MIR2111	AT3G27150	3	Galactose oxidase/kelch repeat superfamily protein
MIR2592	AT4G11040	3	Protein phosphatase 2C family protein
MIR2916	AT1G15780	3	unknown protein
	AT1G50310	3	sugar transporter 9
	AT1G65875	3	pseudogene
	AT1G66120	3	AMP-dependent synthetase and ligase family protein
	AT4G00450	3	RNA polymerase II transcription mediators
	AT5G66710	3	Protein kinase superfamily protein
MIR4414	AT2G04860	3	Tetratricopeptide repeat (TPR)-likesuperfamily protein
MIR4415	AT3G14490	3	Terpenoid cyclases/Protein prenyltransferasessuperfamily protein
MIR5139	AT3G28160	0	transposable element gene
	AT5G27965	1	transposable element gene
	AT4G05360	2	Zinc knuckle (CCHC-type) family protein
	AT5G67460	2	O-Glycosyl hydrolases family 17 protein
	AT1G34470	3	Protein of unknown function (DUF803)
	AT1G64470	3	Ubiquitin-like superfamily protein
	AT1G72800	3	RNA-binding (RRM/RBD/RNP motifs)family protein
	AT2G26430	3	arginine-rich cyclin 1
	AT3G50650	3	GRAS family transcription factor
	AT3G53270	3	Small nuclear RNA activating complex(SNAPc), subunit SNAP43 protein
	AT4G09880	3	unknown protein
	AT5G34450	3	transposable element gene
MIR5256	AT3G47990	3	SUGAR-INSENSITIVE 3
	AT5G47420	3	Tryptophan RNA-binding attenuator protein-like
	AT5G54690	3	galacturonosyltransferase 12
MIR5368	AT5G51130	3	S-adenosyl-L-methionine-dependentmethyltransferases superfamily protein
	AT4G33920	3	Protein phosphatase 2C family protein
MIR5740	AT5G44925	3	transposable element gene
	AT5G50180	3	Protein kinase superfamily protein
MIR6478	AT4G22270	0	Protein of unknown function (DUF3537)
acr-novel2*	AT4G19520	1	disease resistance protein (TIR-NBS-LRR class) family
	AT4G09430	2	Disease resistance protein (TIR-NBS-LRR class) family
	AT4G37190	3	LOCATED IN: cytosol, plasma membrane;
	AT5G45260	3	Disease resistance protein (TIR-NBS-LRR class)

**Figure 5:**
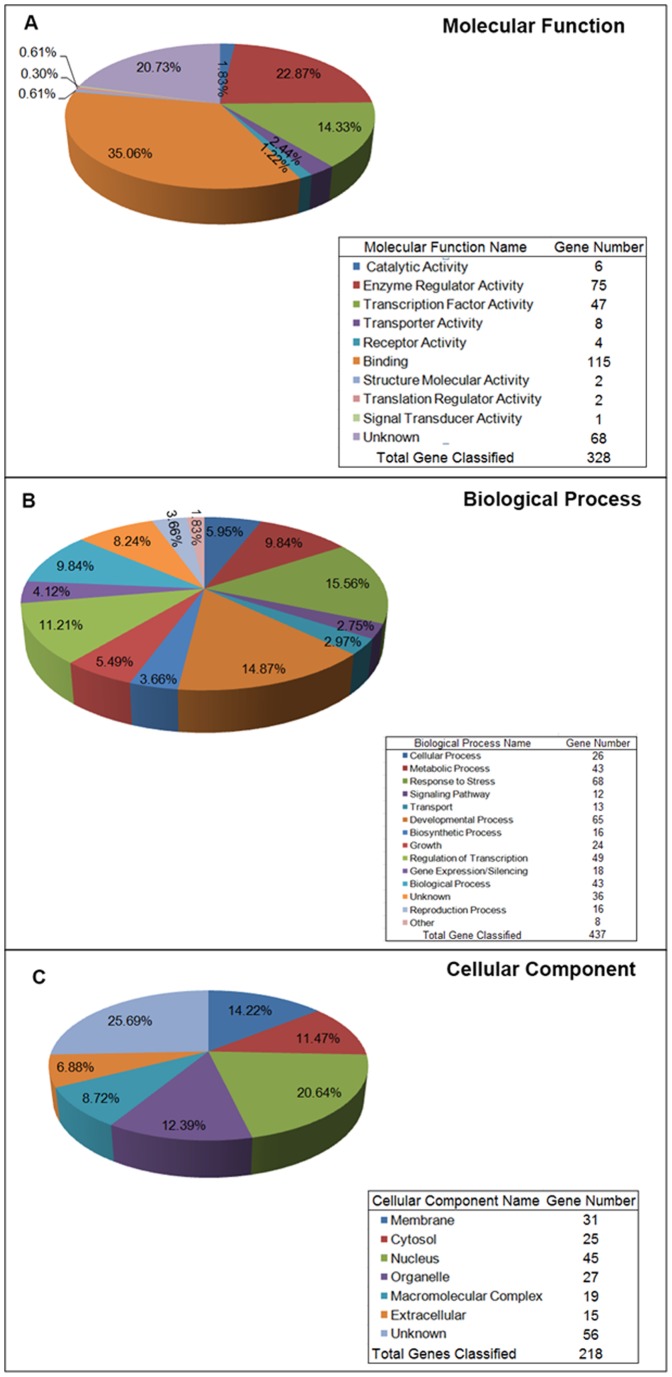
Functional classification of microRNA targets. Gene ontology of the predicted targets for 37 differentially expressed miRNAs. (A) Molecular functions was divided into 10 functional groups. (B) Biological processes was divided into 14 functional groups. (C) Cellular components was divided into seven functional groups.

To describe the gene functions, we classified the potential targets into three categories based on TAIR GO annotations: molecular functions, biological processes and cellular components. For the molecular function category, genes were assigned to eight subcategories (Figure 5A). The GO terms binding (35.06%), enzyme regulator activity (22.87%), and transcription factor activity (14.33%) were the most frequent, and especially RNA polymerase II transcription cofactor activity, which belonged to the transcription factor activity subcategory, is important for miRNA regulation [29]. Biological processes, which included 11 subcategories (Figure 5B), most frequently included GO terms involved in developmental processes (14.87%) and regulation of transcription (11.21%). Five subcategories of cellular components were identified (Figure 5C), of which the most frequent were nucleus (20.64%) and membrane (12.22%). Thus, the high frequency of GO terms associated with developmental processes indicated that many of the miRNAs identified in this study were involved in *A. crassicarpa* organogenesis by regulating molecular functions, biological processes and cellular components.

### Expression Patterns of miRNAs During Organogenesis

**Figure 6:**
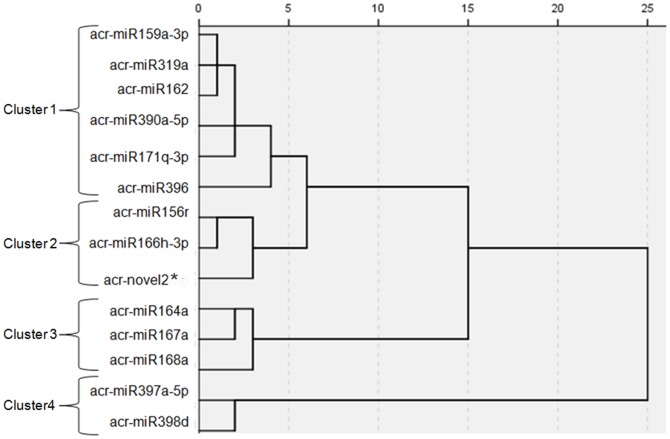
Cluster analysis of expression trends of selected miRNAs. Dendrogram based on expression trends of 14 selected miRNAs during the entire *Acacia crassicarpa* organogenesis process.

To further investigate the role of miRNAs during *A. crassicarpa* organogenesis, 14 miRNAs of known function or high expression counts (one novel and 13 conserved [30]–[43]) (Table 6) were selected for analysis by qRT-PCR [44], which is a reliable method to detect and measure the expression levels of miRNAs in various tissues. The relative quantitative results demonstrated that all selected miRNAs were expressed in different organogenetic tissues and with entirely different expression profiles. On the basis of abundance trends at the six developmental stages, the 14 miRNAs were divided into four clusters through multivariate statistical analysis using general cluster analysis procedure of SPSS Statistics 18.0 (Figure 6).

**Table 6:** miRNAs associated with plant development.

MiRNA	Target gene	Function
miR156	SPL	Flowering control
miR159	MYB	Floral initiation and antherdevelopment; seed germination
miR162	DCLI	miRNA biogenesis
miR164	CUC	Leaf development
miR166	ClassIII HD-ZIPtranscription factor	shoot apical meristem and lateralorganformation
miR167	ARF	Gynoecium and stamen development
miR168	AGO1	miRNA pathway regulation
miR171	SCL	Flower development
miR319	TCP transcription factors	Morphogenesis of leaf
miR390	TAS3 family oftasiRNA-generatingtranscripts	Developmental Time and Pattern
miR396	GRF	Leaf development
miR397	Laccases	Metabolism
miR398	CSD and CytCoxidase:subunit V	Stress respons

**Figure 7:**
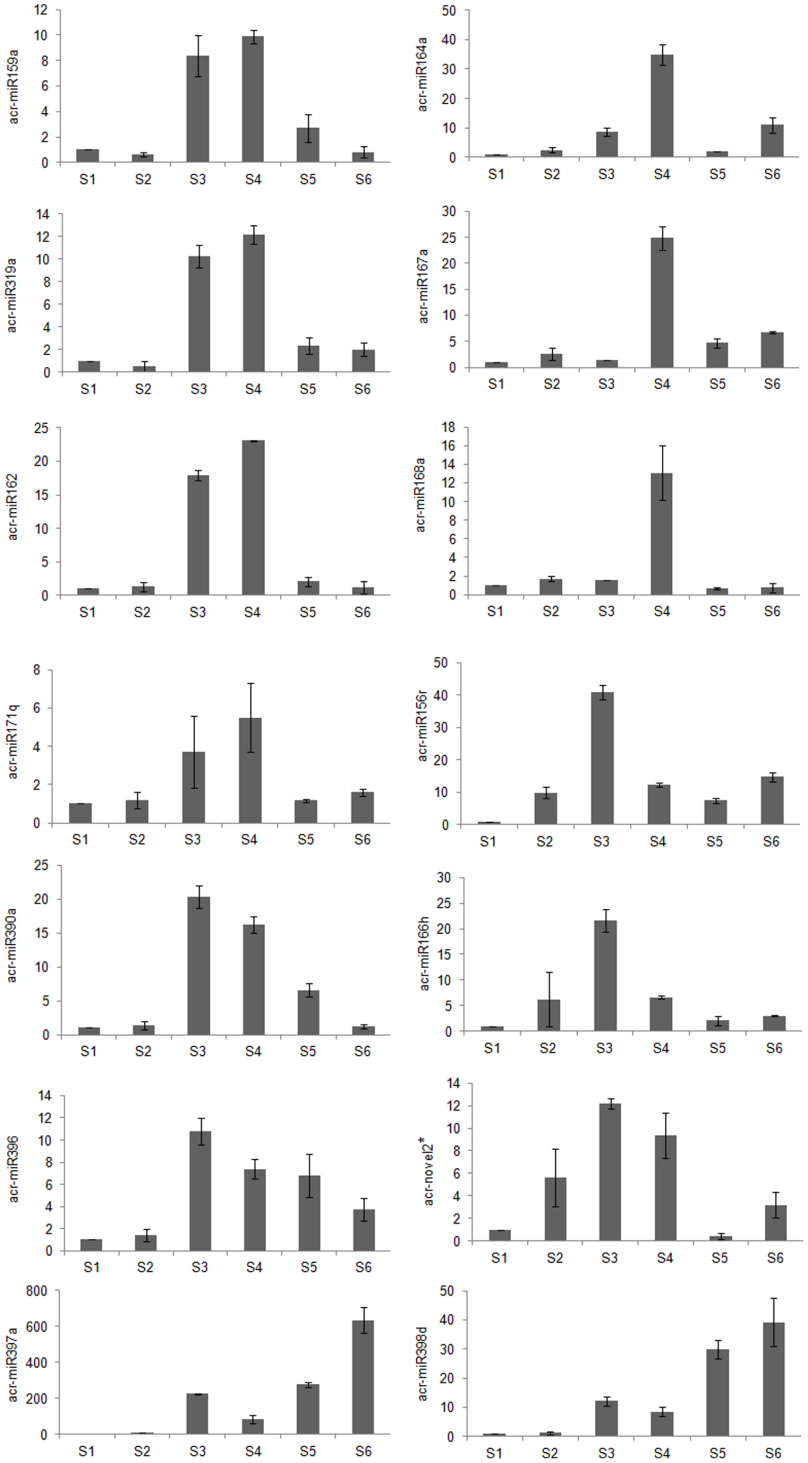
qRT-PCR analysis of relative expression levels of selected miRNAs at six stages of *Acacia crassicarpa* organogenesis. The fold change in gene expression was normalized to controls (mature zygotic embryo) with the 2^−ΔΔCT^ method using 5.8S rRNA as an internal standard. Templates for all miRNAs real-time PCR were 1/20 dilutions of original cDNAs reverse-transcribed from 300 ng miRNA. Each bar shows the mean of triplicate assays.

The first cluster, comprising *acr-miR159a*, *acr-miR319a*, *acr-miR162*, *acr-miR171q*, *acr-miR390a*, and *acr-miR396*, exhibited similar expression patterns and showed a low expression level compared with the other three groups. These miRNAs were barely accumulated during stages S1 and S2 and were induced at stages S3 and S4 when the peak expression level was observed (Figure 7A–F), which implied that their accumulation may be required for embryogenic callus formation. The second cluster, consisting of *acr-miR164a*, *acr-miR167a*, and *acr-miR168a*, showed a stage-specific expression pattern. The expression level peaked during S4 but at the other stages expression was less abundant or undetectable (Figure 7G–I). The cultures were transferred to light at the S4 stage, which suggested these miRNAs might have an important relationship with light. In the third cluster, *acr-miR156*, *acr-miR166*, and acr-novel2* were expressed at varied levels in the different stages. Their major peak in expression was observed at S3, whereas expression at the other stages was relatively lower (Figure 7J–L). The striking differences in expression inferred their function is exerted in different developmental stages. The remaining two miRNAs (*acr-miR397* and *acr-miR398*) showed relatively high expression levels in most of the detected tissues, especially *acr-miR397*. In this group, the miRNAs expression level rose continuously during the successive developmental stages except at S4, and accumulated in S3 and reached their highest levels in the adventitious shoots at S6 (Figure 7M–N), which suggested these miRNAs play a major regulatory role in morphogenesis during advanced differentiation of *A. crassicarpa* adventitious shoots.

The above results concluded that the different miRNAs were indicated to have functions at different developmental stages during *A. crassicarpa* organogenesis.

## Discussion

*In vitro* organogenesis refers to the process involving regeneration of adventitious shoots, adventitious roots or other organs from plant tissue or cell aggregates (callus), which is an important aspect of plant development [45]. Many genes involved in the regulation of organogenesis have been investigated [46], but the molecular mechanisms underlying the process are still not well understood. Previous studies have confirmed that miRNAs play important roles in a variety of developmental processes. However, the role of miRNAs during organogenesis is poorly studied. In the present investigation, we used a high-throughput strategy to perform large-scale cloning and characterization of miRNAs involved in *A. crassicarpa* adventitious shoot organogenesis. We identified 189 known and 7 novel miRNAs from more than 57 miRNA families, and 14 conserved and novel miRNAs were selected for analysis of expression patterns by qRT-PCR. These results provide valuable information on the molecular mechanism of organogenesis in *A. crassicarpa*. Furthermore, target gene prediction and GO annotation demonstrated that putative miRNA targets were involved in a broad variety of regulatory events, including molecular functions, biological processes and cellular components. According to specific stages, the expression patterns of 14 selected miRNAs were observed.

Micropropagation is an important and reliable technique for the production of large quantities of many plant species, particularly as a tool for large-scale plant breeding programs, and is used as a model system to research cell differentiation and development during organogenesis [47]. Changes in miRNA expression during plant somatic embryogenesis has been confirmed in many plants species, such as *A. thaliana* [48], rice [49], Japanese larch [50], Valencia sweet orange [51], *Liriodendron chinense* [52], loblolly pine [53], and longan [54]. However, the function of miRNAs during plant organogenesis is poorly studied. In order to better understand the potential modulation of miRNAs in organogenesis, we studied the expression patterns of miRNAs utilizing the *A. crassicarpa* organogenesis *in vitro* culture system described by Yao *et al* [18]. Combined with a bioinformatic analysis and comparison with miRNA families that are expressed during somatic embryogenesis in other plant species, the results revealed some of the important miRNAs functions related to plant regeneration. Differences in expression pattern were observed among 14 miRNAs analyzed by qRT-PCR. One group of miRNAs (*Acr-miR159a*, *acr-miR319a*, *acr-miR162*, and *acr-miR171q*) were up-regulated at S3 and the expression level peaked at S4 (Figure 7). *MiR159* is a widespread gene present in many plant and animal species [55]. As a highly conserved miRNA in the plant kingdom *miR159* negatively controls gene expression by targeting mainly MYB33 and MYB65 during seed germination and floral development [56]–[57]. In the present study, *acr-miR159a* transcripts accumulated until embryogenic callus was present at stage S3 of *A. crassicarpa* organogenesis, which suggested that *acr-miR159a* might modulate gene expression during embryogenic callus differentiation. This result is similar to the pattern observed in larch [50] and longan [54]. *Acr-miR319a*, which has an extremely similar sequence to that of the *miR159* family, showed similar relative expression levels during *A. crassicarpa* organogenesis [58]. *Acr-miR319a* and *acr-miR159*, showed the same putative target (MYB transcription factors) in the regulation of organogenesis in the present study, which suggests they play similar regulatory roles. The functions of *miR171* and *miR162* are rarely reported. In *A. thaliana*, the potential target of *miR171* is the scarecrow-like (*SCL*) gene family possessing the GRAS domain [59], which is consistent with our findings (Table 5). In our study, these two miRNAs were up-regulated at S3 and S4, indicating that miR171 and miR162 might function in the embryogenic callus, which is consistent with rice somatic embryogenesis [49]. Expression of the remaining miRNAs of the first cluster, *acr-miR390a* and *acr-miR396*, was elevated at S3 then declined, but was maintained at a relatively high level from S3 to S5. *MiR396* is reported to target growth-regulating factor (*GRF*) transcription factors, performing a negative coordinating role in leaf cell proliferation of *A. thaliana* [60]. Overall, members of the first cluster might play regulatory roles in the embryogenic callus stages of *A. crassicarpa* organogenesis.

S4 is a crucial stage during *A. crassicarpa* organogenesis when buds are induced and began to turn green, and are ready to develop into adventitious shoots. miRNAs of the second cluster, consisting of *acr-miR164a*, *acr-miR167a*, and *acr-miR168a*, exhibited stage-specific expression, which indicated that they may have stage-specific functions during bud formation. These three miRNAs were expressed at low or undetectable levels at all stages except S4, during which expression peaked. In *Arabidopsis*, *miR164* mainly controls the NAM/ATAF/CUC (*NAC1*) domain-transcription factor family. NAC1 is involved in transitions in auxin signalling, and facilitates growth of lateral roots [61]. CUC regulates organ separation from fasciculate buds during embryogenesis. In the present study, the same putative target (NAC-domain transcription factor) implied that *miR164* has the same function in *A. crassicarpa*. *MiR168* targets the *AGO1* gene, which is involved in plant development by feed-back regulation. The expression patterns of *acr-miR164a* and *acr-miR168a* were consistent with results reported for citrus after callus is cultured in the light [51]. However, our findings were inconsistent with the pattern observed in larch, in which *miR168* was not expressed at a notably high level when callus began to turn green [50]. These conflicting results suggest that the function of *miR168* might vary between species. Aux/IAA and ARF, the putative targets of *miR167*, are two important protein families that respond to auxin signal. Ru *et al.* [62] suggested that the high abundance of ARF8 led to the low expression level of *miR167*. These results imply the target of *miR167* conservative property. In conclusion, this cluster of miRNAs may function in the bud formation stages and response to light in *A. crassicarpa* organogenesis.

The important events that occur during *A. crassicarpa* adventitious shoot organogenesis from a zygotic embryo involve redifferentiation. In rice [49], *miR156* is important in the transition from undifferentiated to differentiated callus during somatic embryogenesis by targeting SPL genes. Zhang *et al.* [50] and Wu *et al.* [51] reported that SPL controls the somatic embryo induction process. *SPL* genes also have other regulatory roles in different biological processes, such as induction of the floral transition and consequent shortening of the vegetative phase [63], and regulation of the juvenile to adult transition during plant development [64]. In the present study, *acr-miR156r* was accumulated at S3 while the zygotic embryo is differentiating into embryogenic callus during organogenesis. As shown in Figure 7, the abundance of the other members of the third cluster (*acr-miR166h* and acr-novel2*) also increased continuously until S3,but exhibited very low or undetectable expression levels during S4–S6 and especially at S5. Given that these three miRNAs showed similar expression patterns, we hypothesize that they modulate redifferentiation during induction of organogenesis in *A. crassicarpa* as the fatal genes.

With regard to the fourth cluster, relatively high expression of *acr-miR397* and *acr-miR398* was observed. *MiR397*, for which a target gene of laccase is implicated, is associated with lignin biosynthesis and primary cell wall [65]–[66]. The detection of *miR397* and corresponding targets occurs during the regulation of lignification and thickening of the cell wall in secondary cell growth in rice, larch and citrus [49]–[51]. *MiR398* is known to target Cu/Zn superoxide dismutases (CDSs), which are associated with stress response [43]. In the present study, the levels of *acr-miR397* and *acr-miR398* increase continually until S6, at which stage the expression level peaked, except for a slight decline at S4. Zygotic embryos undergo differentiation at stages S1–S3 until the formation of buds at S4, which suggests that laccase, the target gene of *miR397*, regulates lignification and cell wall thickness during organogenesis. The decrease in expression level of miR397 at S4, when a cluster of adventitious shoots had developed, is attributed to negative modulation of the formation of thickened cell walls for adventitious shoots.

In summary, a global analysis of miRNAs expression during *A. crassicarpa* adventitious shoot organogenesis was carried out. The results of a bioinformatic analysis and experimental tests revealed putative regulatory functions for the miRNAs in *Acacia crassicarpa* organogenesis. These findings provide important information for deep sequencing research of miRNAs and future large-scale propagation and breeding of leguminous trees.
